# Monte Carlo simulation of 6‐MV dynamic wave VMAT deliveries by Vero4DRT linear accelerator using EGSnrc moving sources

**DOI:** 10.1002/acm2.13090

**Published:** 2020-11-21

**Authors:** Maryam Rostamzadeh, Yoshitomo Ishihara, Mitsuhiro Nakamura, I. Antoniu Popescu, Ante Mestrovic, Ermias Gete, Roberto Fedrigo, Alanah Mary Bergman

**Affiliations:** ^1^ Department of Physics and Astronomy University of British Columbia Vancouver BC Canada; ^2^ Japanese Red Cross Wakayama Medical Center Wakayama Japan; ^3^ Graduate School of Medicine Kyoto University Kyoto Japan; ^4^ Medical Physics Department BC Cancer‐Vancouver Vancouver Canada

**Keywords:** BEAMnrc, DOZXYZnrc, dynamic wave arc, EGSnrc, Monte Carlo, Vero4DRT

## Abstract

The commissioning and benchmark of a Monte Carlo (MC) model of the 6‐MV Brainlab‐Mitsubishi Vero4DRT linear accelerator for the purpose of quality assurance of clinical dynamic wave arc (DWA) treatment plans is reported. Open‐source MC applications based on EGSnrc particle transport codes are used to simulate the medical linear accelerator head components. Complex radiotherapy irradiations can be simulated in a single MC run using a shared library format combined with BEAMnrc “source20.” Electron energy tuning is achieved by comparing measured vs simulated percentage depth doses (PDDs) for MLC‐defined field sizes in a water phantom. Electron spot size tuning is achieved by comparing measured and simulated inplane and crossplane beam profiles. DWA treatment plans generated from RayStation (RaySearch) treatment planning system (TPS) are simulated on voxelized (2.5 mm^3^) patient CT datasets. Planning target volume (PTV) and organs at risk (OAR) dose–volume histograms (DVHs) are compared to TPS‐calculated doses for clinically deliverable dynamic volumetric modulated arc therapy (VMAT) trajectories. MC simulations with an electron beam energy of 5.9 MeV and spot size FWHM of 1.9 mm had the closest agreement with measurement. DWA beam deliveries simulated on patient CT datasets results in DVH agreement with TPS‐calculated doses. PTV coverage agreed within 0.1% and OAR max doses (to 0.035 cc volume) agreed within 1 Gy. This MC model can be used as an independent dose calculation from the TPS and as a quality assurance tool for complex, dynamic radiotherapy treatment deliveries. Full patient CT treatment simulations are performed in a single Monte Carlo run in 23 min. Simulations are run in parallel using the Condor High‐Throughput Computing software^1^ on a cluster of eight servers. Each server has two physical processors (Intel Xeon CPU E5‐2650 0 @2.00 GHz), with 8 cores per CPU and two threads per core for 256 calculation nodes.

## 1 INTRODUCTION

**Fig. 1:**
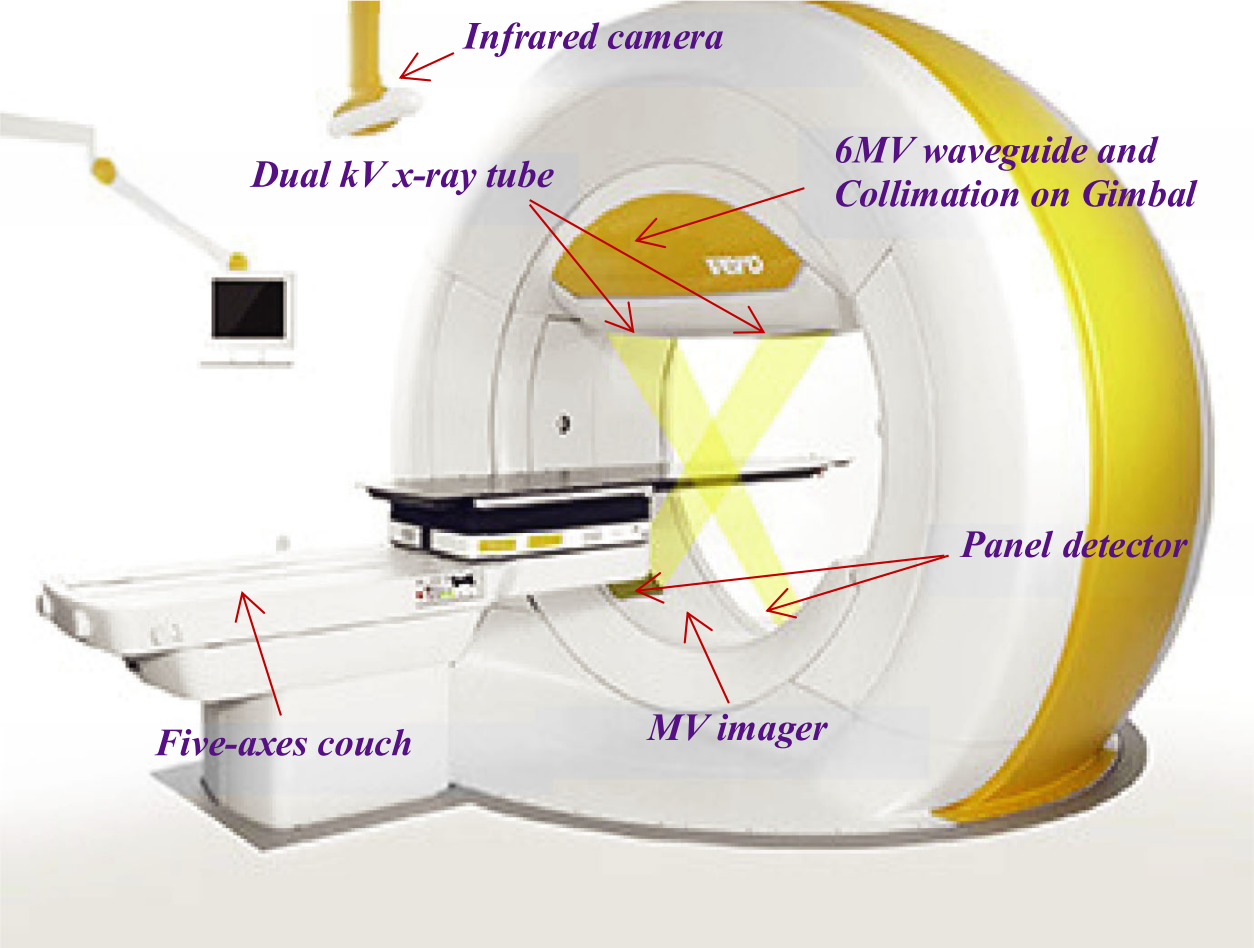
Vero4DRT.

The Brainlab‐Mitsubishi Vero4DRT platform (Brainlab AG, Munich, Germany) is a 6‐MV‐medical linear accelerator with an O‐ring gantry design.^1^, ^2^, ^3^, ^4^ It has two axes of beam rotation; longitudinal — achieved with an O‐ring gantry, and vertical — achieved with a floor‐ring rotation (Fig. 1).

Unlike conventional linear accelerators, noncoplanar beam angles are achieved without moving the patient couch (the unit moves around a stationary patient). The Vero4DRT is a dedicated stereotactic ablative radiotherapy (SABR) unit capable of delivering conventional three‐dimensional conformal radiation therapy (3DCRT) beams, conformal arcs, static field intensity‐modulated radiotherapy (IMRT) beams, and VMAT arcs. In addition, the Vero4DRT has a unique clinical delivery mode called dynamic wave arc (DWA) which employs noncoplanar VMAT trajectories created by enabling simultaneous motion of the gantry and floor ring about the two axes of rotation.^5^, ^6^, ^7^ The unit is equipped with integrated, dual‐orthogonal kV image guidance systems (ExacTrac, Brainlab, Munich, Germany) and is cone‐beam CT (CBCT) capable. The entire waveguide and collimation assembly is mounted on a 2D‐gimbal which provides real‐time, respiratory motion‐correlated dynamic tumor tracking (DTT) capability. The unit only has one static secondary collimator (jaw) field size which is set to 15 × 15 cm^2^ at source to axis distance (SAD) of 100 cm. All beam shaping is achieved with a low transmission (~0.11–0.13%)^4^, ^8^ multileaf collimator (MLC). This MLC has 30 pairs of 5 mm width (at isocenter) tungsten leaves.^8^

The Vero4DRT has been applied to various clinical scenarios including lung, liver, pancreas, breast, prostate, bone, and brain.^9^, ^10^, ^11^, ^12^, ^13^

Monte Carlo modeling is a useful option for providing secondary dose calculation verification and has the added benefit of providing “gold standard” dose calculations in inhomogeneous materials.^14^ It has been shown that MC models can provide useful information on dose calculation algorithm accuracy for smaller treatment fields (e.g., SABR or stereotactic radiosurgery type beam deliveries).^15^, ^16^, ^17^ Ishihara et al. report on a Monte Carlo model for the Vero4DRT and its low‐transmission MLC using the EGSnrc “VARMLC” component module.^18^ This model was reported for static‐field beam geometries.

With “moving” sources available (e.g., EGSnrc “source 20 and 21”), Monte Carlo can efficiently model complex, noncoplanar dynamic beam trajectories.^19^ In addition, there is potential to model the dynamic tumor tracking respiratory‐correlated motions on 4DCT datasets.^20^, ^21^ The goal of this study is to further develop the Ishihara et al. Vero4DRT static beam linac model and introduce dynamic motions into the simulation (e.g., simultaneous MLC, gantry and ring) to allow for efficient dose calculations on patient CT sets of the DWA beam delivery delivered by the Vero4DRT.

## 2 MATERIALS AND METHODS

### 2.A General Vero4DRT geometry for Monte Carlo simulation

**Fig. 2:**
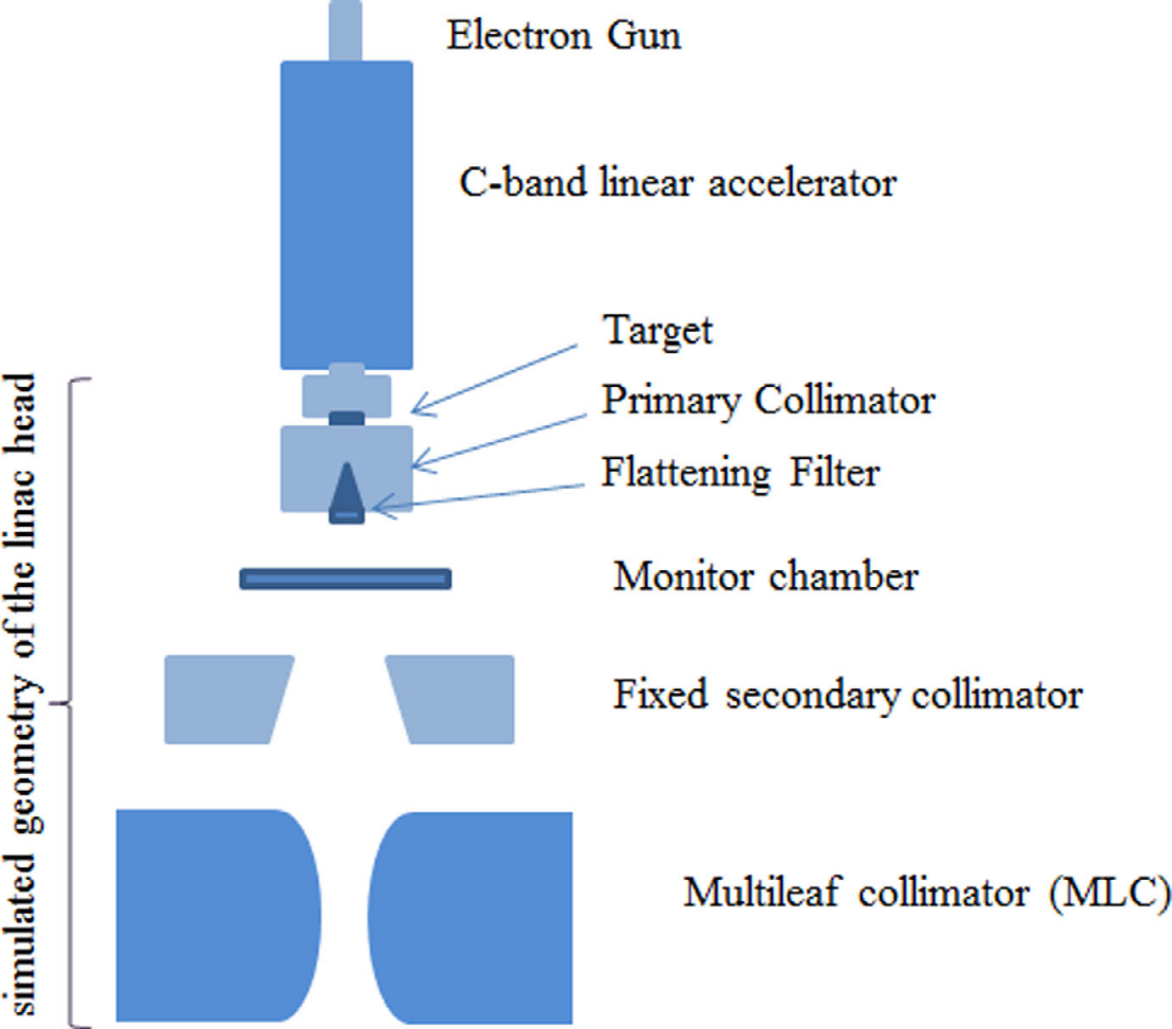
Geometric schematic of the x‐ray head and multileaf collimator for Vero4DRT. Note: the entire system is mounted on a movable two‐dimensional gimbal for dynamic tumor tracking. The secondary collimator (jaws) is fixed at one 15 × 15 cm^2^field size.

The EGSnrc/BEAMnrc code (NRC, Ottawa, Canada, v2018) is used to create the virtual x‐ray photon beam.^22^, ^23^ This code generates 3D models of a linear accelerator treatment head. The components of the Vero4DRT medical accelerator and treatment head are shown in Fig. 2.

In BEAMnrc, features of the incident electron beam geometry onto the target are controlled in a module called “source” which offers several options to the operator. “ISOURC19” was used in this model. This is an “Elliptical Beam with Gaussian Distributions in X and Y.” The electron beam has parallel edges, with direction cosines specified by the user. The incident energy and the FWHM values for the incident electron beam can be varied until the simulated dose to water matches experimental commissioning measurements.

Photons and electrons produced in the tungsten target are transported through each component module of the linac head. The linac head components including target, primary collimator, flattening filter, ionization chambers, secondary collimator jaws, and MLC were modeled in a Monte Carlo environment (BEAMnrc) based on manufacturer specifications.^8^, ^18^ The composition of the materials and alloys, mass densities, position, dimensions, and shape of defining surfaces of the components, plus the properties of motion are all defined in detail within these modules.

In this study, a phase space plane (file describing particle type, energy, directional vectors, and location of last interaction) is defined just above the MLC and below the static secondary collimator (jaw) at a distance of 35.2 cm from the target. It is important to note that the Vero4DRT only has one static secondary collimator field size which is set to 15 × 15 cm^2^ at source to axis distance (SAD) of 100 cm. This phase space represents the nonpatient‐specific geometry of the linear accelerator. The phase space file is generated using 1.5 × 10^9^ initial electrons incident on the target, which are used for subsequent simulations through the MLC. This number of electrons incident on the target reduces the overall uncertainty in the phase space in BEAMnrc and reduces the need to recycle particles in order to obtain adequate statistics. The photon cutoff energy (PCUT) is set to 0.01 MeV, and the electron cutoff energy (ECUT) is set to 0.521 MeV for all simulations. The cutoff energy is a variance reduction technique which helps to reduce the calculation time. This technique disregards any possible future interactions of the photon and electron at this cutoff energy so must be applied with care. The accuracy of result increases with decreasing cutoff energy, however, the calculation time increases.

By capturing a phase space file below the static components, the user can reuse it to transport particles through the moving (patient‐specific) parts of the linac (i.e., MLC).

Radiation beams generated by BEAMnrc can be directed onto a voxelized phantom of CT patient data from patient‐specific gantry and ring angles and radiation doses are scored using DOSXYZnrc codes.^24^ In this study “source 20” was used^19^ which allows the user to simulate dynamic motion of the virtual phase space source relative to the patient geometry or phantom.

The goal is to simulate enough histories to achieve <2% uncertainty in the patient or phantom voxel dose value. Simulations are run in parallel using the Condor High‐Throughput Computing software^25^ on a cluster of eight servers, under the Red Hat Enterprise Linux (release 6.4) operating system. Each server has two physical processors (Intel Xeon CPU E5‐2650 0 @ 2.00 GHz), with 8 cores per CPU and two threads per core for a total of 256 calculation nodes.

### 2.B MLC modeling

All dynamic beam shaping is achieved with a low‐transmission (0.11–0.13% average)^4^, ^8^ 60‐leaf, tungsten MLC. The MLC Monte Carlo physical model parameters were the same as those used by Ishihara et al.^18^ The MLC has 30 pairs of 5 mm width (at isocenter) tungsten leaves with a maximum field size of 15 × 15 cm^2^. Leaf height and length are 11 and 26 cm, respectively. Each leaf has a circular end, with a radius of curvature of 37 cm and tongue‐groove design (5.5 cm groove height).^8^ Unlike the Ishihara et al. model which employed the BEAMnrc VARMLC module,^18^ this study employs the SYNCVMLC component module. The SYNCVMLC component module allows synchronization of leaf opening with any other synchronized component in the linac by combining with source 20 (synchronized phase space source used in DOSXYZnrc) developed by Lobo and Popescu.^19^ DOSXYZnrc calls for particles transported through the BEAMnrc shared libraries that model the dynamic MLC.

The simulation of simultaneous multiple fields, dynamic gantry and ring rotations and dynamic MLC shapes can now be performed in a single MC simulation.

### 2.C Setup and measurement

#### 2.C.1 Benchmarking open fields

Percent depth doses and crossplane/inplane profiles were measured for field sizes ranging from 1 × 1 to 15 × 15 cm^2^ during the commissioning of the linac and treatment planning system. Dose measurements were performed in water phantom (Blue Phantom, IBA Dosimetry, USA) for the 6‐MV photon beam. A CC13 (0.13 cm^3^) volume ion chamber (IBA Dosimetry, USA) was used for field sizes larger than 4 × 4 cm^2^. For smaller field sizes (<4 × 4 cm^2^), a CC01 (0.01 cm^3^) volume (IBA Dosimetry, USA) ion chamber and microDiamond detector (PTW, Germany) were used. PDDs and profiles were acquired at SSD 90 cm per Brainlab commissioning protocols.

A Monte Carlo representation of the water phantom with dimensions of 30 × 30 × 30 cm^3^ and a voxel size of 0.2 × 0.2 × 0.2 cm^3^ was created. Percentage depth doses and beam profiles in this water phantom are simulated using DOSXYZnrc. A total of 9 × 10^8^ histories (called by DOSXYZnrc to calculate the dose in phantom) was used for the calculations to achieve statistical uncertainties of <2%.

The resulting dose distribution is compared to measured dose data. The tuning of the electron beam energy incident on the target is performed by comparing calculated and measured PDD curves. 6 MV is the nominal energy for the Vero4DRT. The initial electron energy for 6 MV differs for each linac model. It depends on the construction and materials of the target, flattening filter, and other components.

In this study, comparison of PDD curves (measured vs simulated) is used to define this value. Five different incident electron energies were assessed in an effort to match the simulated PDD curves to measurement. The incident electron beam energy was varied from 5.5 to 6.5 MeV in steps of 0.1 MeV (based on the methodology from Ishihara et al.^18^). The best match (found by minimizing the percent difference between measurement and simulation for the descending part of the PDD) determined the optimum energy of electrons incident on the target used in the Monte Carlo simulation. Using this method, the electron energy was determined to be 5.9 MeV. The best match (found by minimizing the percent difference between the descending part of the PDD) determined the optimum energy of electron beam. Local percent differences between simulation and measurements results are reported. The electron beam FWHM gaussian width was varied from 1.5 to 2.2 mm in steps of 0.1 mm and simulated beam profiles were compared to measurement (normalized to 100% at central axis dose). The electron beam gaussian width was optimized for a 10 × 10 cm^2^ field size at depth of d = 10 cm (found by minimizing the percent difference in low dose gradient regions of the profiles).

Once the beam energies and electron FWHM gaussian width were optimized, PDDs and beam profiles for field sizes ranging from 1 × 1 to 15 × 15 cm^2^ were compared and assessed for agreement.

#### 2.C.2 Absolute dose conversion

The calibration conditions on the clinical Vero4DRT is such that 1 MU will deliver 1 cGy to the patient at depth of 1.5 cm for a 10 × 10 cm^2^ field size at SAD of 100 cm. The radiation beam is directed onto a water phantom and dose distribution calculated using DOSXYZnrc. The calibration geometry for the virtual linac is a 10 × 10 cm^2^ field, SAD of 100 cm and an isocenter depth of 10 cm. The raw dose from the DOSXYZnrc Monte Carlo simulation is in units of “dose per particle incident on the target.” The Monte Carlo “dose per particle hitting the target” is converted to absolute doses by assigning a known dose for a known Monitor Unit setting to a point in phantom at a depth of 10 cm. This dose point at depth of 10 cm is converted to the calibration dose at d_max_ (1.5 cm) by applying a measured tissue maximum ratio (TMR) factor of 0.772. Once this calibration has been established, the “Gy/Monte Carlo dose” factor can be applied to all voxels. For the calibration conditions, 1 MU corresponds to 6.2258 × 10^15^ simulated electrons incident on the target.

The Vero4DRT unit only has one static secondary collimator (jaw) field size which is set to 15 × 15 cm^2^ at source to axis distance (SAD) of 100 cm. All beam shaping is achieved with the MLC. Corrections for field size dependence of the monitor chamber dose, as reported by Popescu et al.,^26^ are not necessary when converting Monte Carlo dose to absolute dose for the Vero4DRT. The effect of radiation backscattered from the MLC to the monitor chamber was found to be minimal and can be ignored. This is confirmed by comparing absolute dose PDDs and profiles for various field sizes.

#### 2.C.3 MLC transmission

The Vero4DRT has an amorphous silicon electronic portal imaging detector (EPID) (Perkin Elmer, Santa Clara, CA) which is mounted on ring. The EPID’s distance from source is 221.2 cm and it is 0.4 mm per pixel at the detector plane.

The average MLC transmission value is the ratio of closed leaf doses to open field doses, averaged across the leaf bank at the center of the field (x = 0 cm). This ratio was measured using both a CC13 ion chamber (1000 MU delivered, open field size 10 × 10 cm^2^, d = 1.5 cm) and the EPID (5 MU delivered, open field size 15 × 15 cm^2^). Percent transmission values are compared to MC simulation on a cube phantom (0.25 × 0.25 × 0.25 cm^3^ voxel size) at depth of 1.5 cm, field size 10 × 10 cm^2^. A total of 9 × 10^8^ histories was simulated in DOSXYZnrc for both the closed MLC and open field simulations.

#### 2.C.4 Static MLC bar pattern

**Fig. 3:**
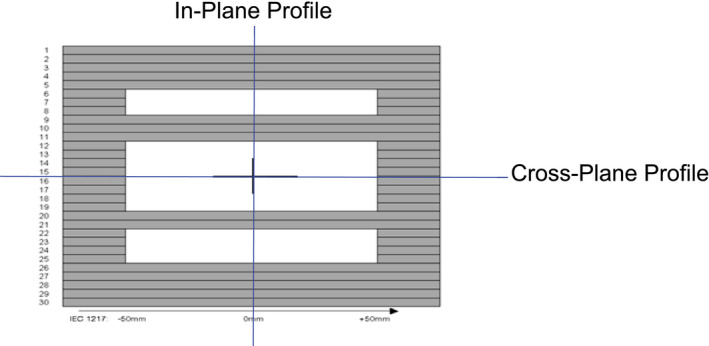
Multileaf collimator (MLC) field setup for inplane and crossplane profile measurement. MLC leaf width is 5 mm at isocentre, 100 cm from the photon source.

Measured and Monte Carlo simulated absolute doses were created for a static MLC pattern (standard Brainlab iPlan commissioning pattern) as shown in the Fig. 3. The inplane and crossplane dose distributions are compared at 1.5 cm depth (SAD 100 cm) in water phantom. The MC simulation is performed on a water phantom geometry with a 0.25 × 0.25 × 0.25 cm^3^ voxel size.

### 2.D Dynamic Wave Arc (DWA) verification

After completion of the validation of the phase‐space data for commissioning fields, Monte Carlo calculations were performed on a dynamic, noncoplanar trajectory‐VMAT (DWA) treatment plan that was generated by the RayStation (RaySearch, Sweden, v 7.0) treatment planning system (TPS) which employs the collapsed cone convolution (CCC) algorithm.^27^ A calculation grid size of 2.0 mm was used for all treatment plans. The MLC configuration parameters in RayStation were 0.0 cm for the leaf tip width, and 0.1% for transmission.

Institutional Research Ethics Board approval was obtained for this validation study.

**Fig. 4:**
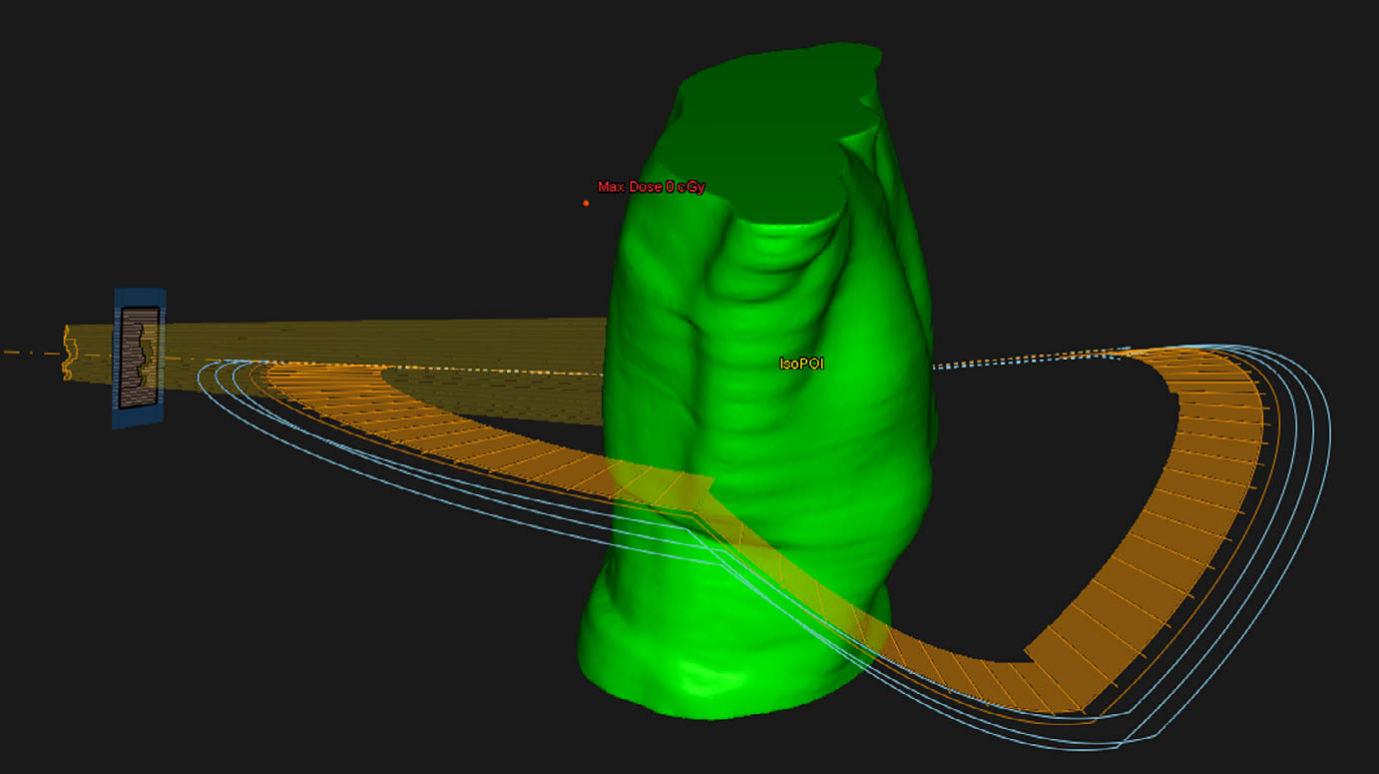
Liver stereotactic ablative radiotherapy treatment plan using dynamic, non‐coplanar volumetric modulated arc theraphy trajectories (dynamic wave arc).

A complex liver stereotactic ablative radiotherapy (SABR) VMAT treatment plan using dynamic, noncoplanar trajectories (DWA) was simulated (Fig. 4). Doses were prescribed to cover 95% of the planning target volume (PTV) with a prescription dose of 54 Gy, delivered in three fractions.

The entire Monte Carlo simulation is scripted^28^ such that the user only has to provide the initial DICOM files for the plan parameters, dose matrix, CT and structure set from RayStation. The script automatically generates the patient‐geometry MC phantom (using ctcreate), creates all input files required by the BEAMnrc and DOSXYZnrc simulations, and launches the distributed simulation. Once a Monte Carlo 3D dose distribution is created on the patient geometry, additional processing is optionally applied such as a 3D denoising filter (based on Savitzky–Golay formalism).^29^, ^30^ The conversion to absolute dose is applied and a TPS‐compatible DICOM dose matrix containing the Monte Carlo dose is generated (for import back into the TPS to provide dose/DVH comparisons).

The DICOM CT datasets were converted to a Monte Carlo phantom using a voxel size of 2.5 mm^3^. DOSXYZnrc simulation parameters were set to achieve statistical uncertainty <2% in the dose calculation, (~9 × 10^8^ histories). The MC calculated doses are imported into the TPS for dose distribution comparison purposes. On the patient CT datasets, target coverage (PTV) and organ‐at‐risk dose volume constraints are compared.

The DWA plan was also calculated on an in‐house, cylindrical, uniform density quality assurance phantom (26.8 cm in diameter consisting of Hi Impact polystyrene). CCC calculated and Monte Carlo simulated distributions are compared by means of a 3D gamma comparison (3% Dose Difference (relative to D_max_)/3 mm Distance‐To‐Agreement /30% threshold).

The TPS dose distribution is compared to the MC‐generated dose distribution. A PASS status for the cylinder QA is met if more than 95% of the data points have a gamma value <1.

## 3 RESULTS

### 3.A Benchmarking open fields

#### 3.A.1 Percent depth dose

**Fig. 5:**
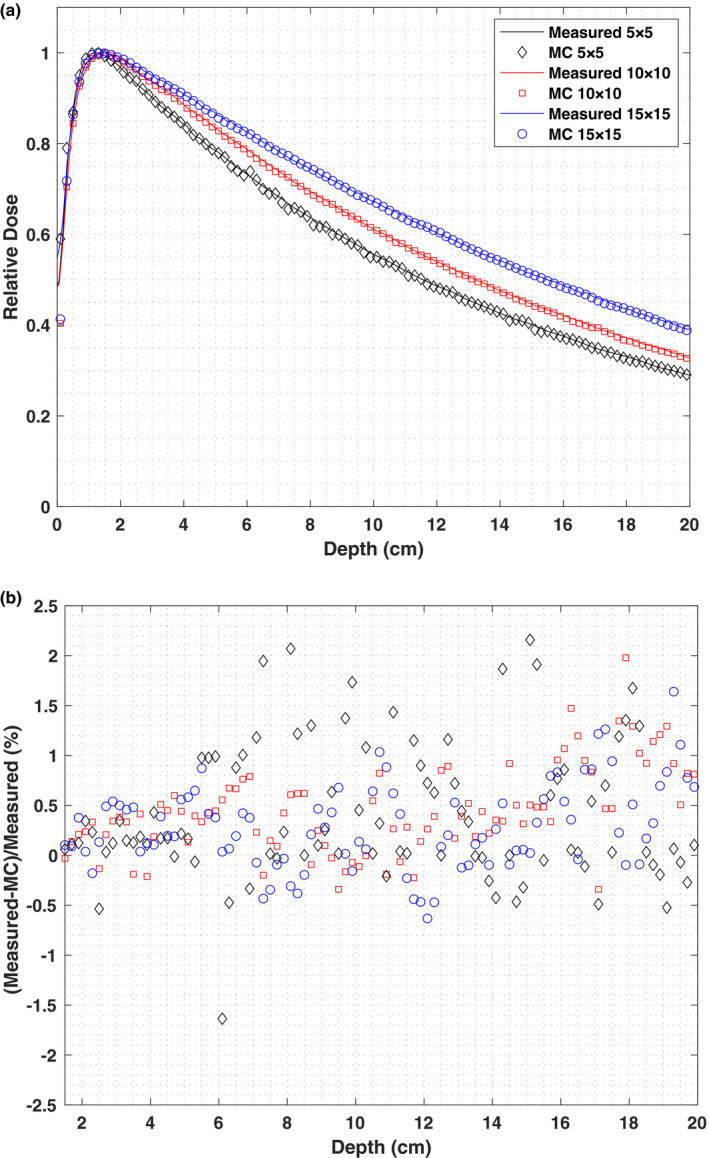
(a) Comparison of simulated and measured percentage depth dose (PDD) for field size of 5 × 5, 10 × 10 and 15 × 15 cm^2^at SSD = 90 cm. Electron beam nominal energy at the target was 5.9 MeV. Doses are normalized relative to the dmax dose. (b) PDD differences between Monte Carlo.

The incident electron energy on the target affects the Monte Carlo simulated depth dose curves in water phantom. Higher photon beam energies will shift the PDDs to deeper depth. The dose differences between the MC simulation and the water‐tank measurements were minimized with an incident electron energy of 5.9 MeV. Monte Carlo simulated PDD curves for the Vero4DRT 6 MV beam are compared to measured doses, as shown in Fig. 5(a). Three field sizes of 5 × 5 cm^2^, 10 × 10 cm^2^, and 15 × 15 cm^2^ are shown. Doses are normalized relative to the d_max_ dose. The statistical uncertainty is less than 2% in the PDD calculation and error bars are equal or smaller than symbol sizes. The differences between measurement and Monte Carlo simulations are smaller than 2.15% for the descending part of PDDs [Fig. 5(b)].

#### 3.A.2 Beam profiles

The beam profiles for a 1 × 1, 5 × 5 and 10 × 10 cm^2^ open field size (SSD = 90 cm) for 5.9‐MV Monte Carlo photon beam were compared at depth of 10 cm in water phantom. The calculated beam profile with electron beam gaussian width of 1.9 mm agreed the closest with measurements and the resulting field metrics are shown in Table 1. The differences between nominal and MC calculated field sizes were within 1.5 mm. The differences between measured and MC calculated field sizes were within 0.4 mm. The differences between measured and MC calculated penumbra size were within 1 mm.

**Table 1:** Comparison of measured and calculated distances to the field edges (X50% and Y50%) and beam penumbras (X20%−X80%) and (Y20%−Y80%) for field size 1 × 1, 5 × 5 and 10 × 10 cm^2^ at depth of 10 cm and SSD 90 cm.

Field size (cm^2^)	Crossplane				Inplane			
	X50% (cm)		X20%‐X80% (cm)		Y50% (cm)		Y20%‐Y80% (cm)	
	Measured	MC	Measured	MC	Measured	MC	Measured	MC
1 × 1	1.12	1.15	0.32	0.35	0.97	1.01	0.31	0.32
5 × 5	5.12	5.12	0.61	0.57	4.97	4.98	0.58	0.59
10 × 10	10.15	10.14	0.69	0.59	10.03	10.03	0.67	0.59

**Fig. 6:**
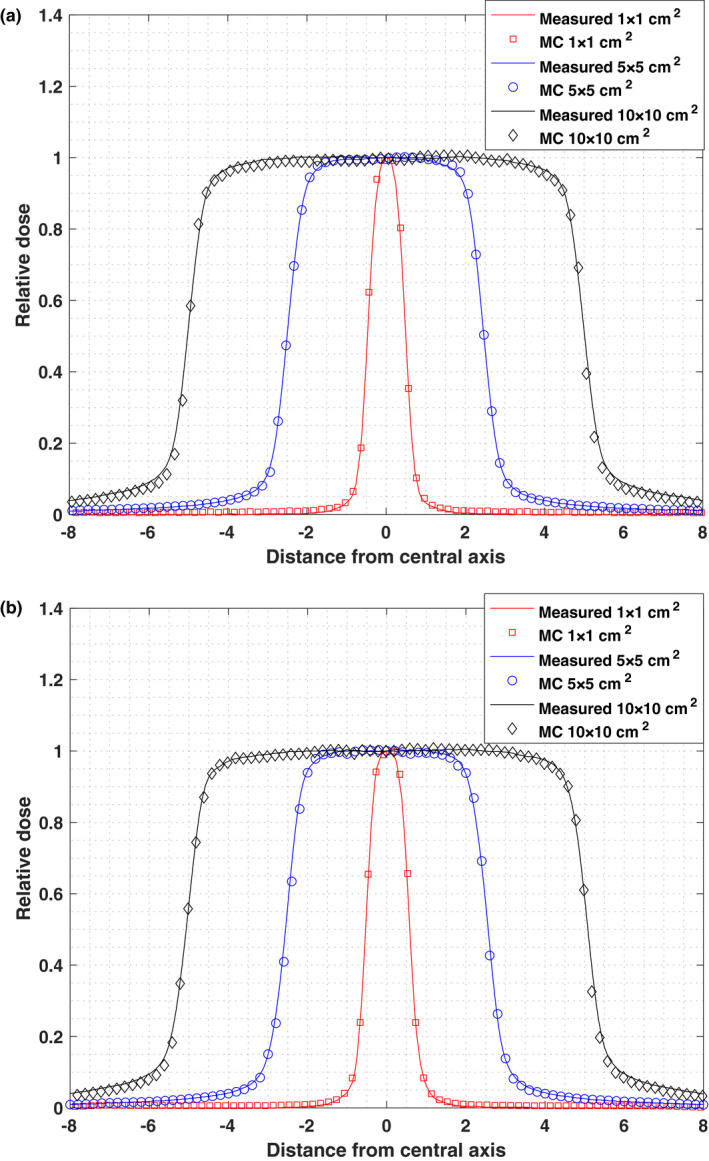
(a) Inplane and (b) Crossplane profile of simulated dose and measured dose at depth of 10 cm at SSD 90 cm, field size 1 × 1, 5 × 5 and 10 × 10 cm^2^. Electron beam FWHM is 1.9 mm. Doses are normalized to central axis value.

Monte Carlo simulated inplane and crossplane beam profile curves for field sizes of 1 × 1, 5 × 5 and 10 × 10 cm^2^ of the Vero4DRT 6MV beam are compared to measured doses, as shown in Fig. 6. Doses are normalized to the central axis value. The statistical uncertainty is <2% in the PDD dose calculation and error bars are equal or smaller than symbol sizes.

#### 3.A.3 MLC transmission (average inter/intraleaf leakage)

**Fig. 7:**
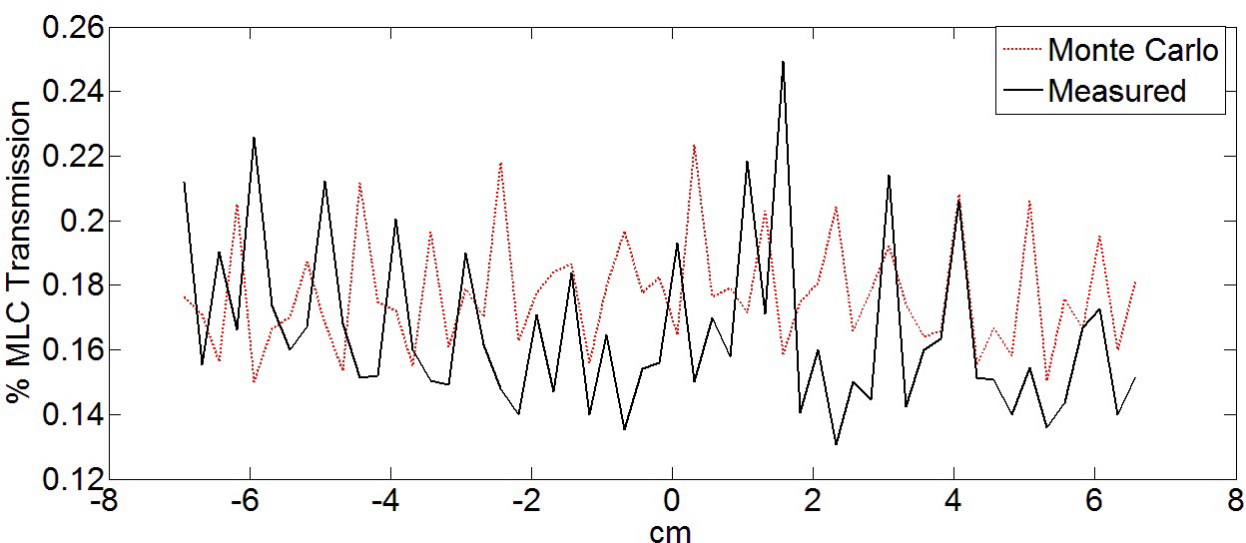
Leaf leakage percent ratio to determine the transmission properties of the multileaf collimator of the Vero4DRT. Profile is perpendicular to leaf motion (across the leaves). EPID‐measured transmission (solid line) and Monte Carlo simulated transmission (dotted line) are shown.

The EPID measured and Monte Carlo simulated leakage along the path perpendicular to MLC travel is shown in Fig. 7. The average EPID measured transmission value is 0.17%. The MC calculated average transmission is 0.18%. The simulation has been done on a cube phantom with 0.25 × 0.25 × 0.25 cm^3^ voxels size and the reason that peaks are not 0.5 cm (the leaf width) apart is because of the pixel volume averaging effect.

#### 3.A.4 Static MLC bar pattern

**Fig. 8:**
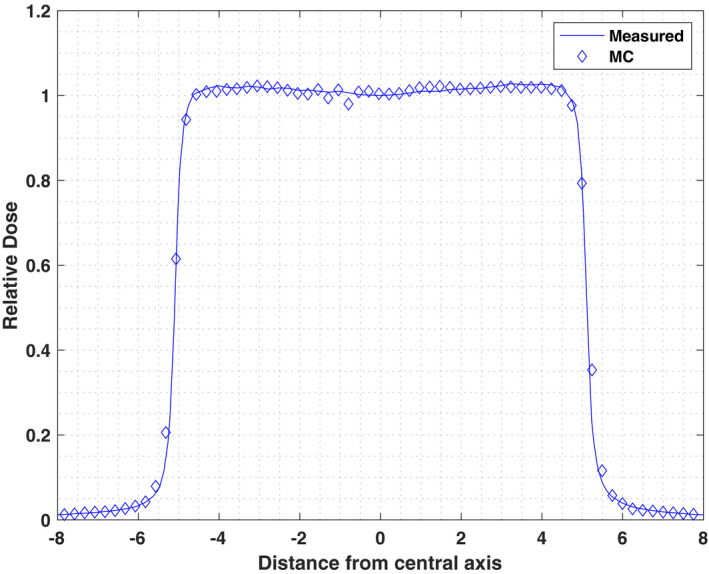
Crossplane profiles of the simulated and measured doses for multileaf collimator Bar Pattern at SSD = 100 cm at depth = 1.5 cm.

**Fig. 9:**
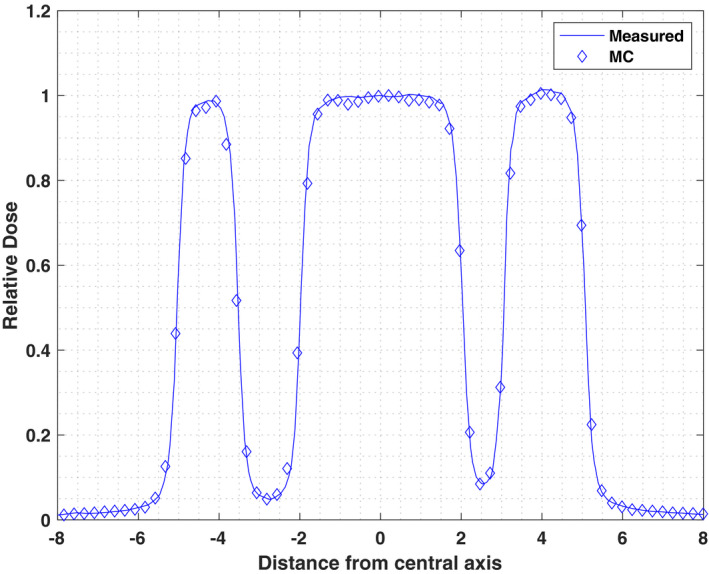
Inplane profiles of the simulated and measured doses for multileaf collimator Bar Pattern at SSD = 100 cm at depth = 1.5 cm.

A MC simulation through a standard Brainlab commissioning MLC bar pattern was performed for SSD of 100 cm in water phantom with 9 × 10^8^ histories simulated in DOSXYZnrc. Calculated dose profiles in x and y direction were acquired and compared to measured water tank data. Figures 8 and 9 illustrate the comparison of crossplane and inplane profiles measured at depth of 1.5 cm. Each beam profile is normalized to its value along the central axis. For low dose gradient regions (±5% zone from max or min value) the differences between measurement and Monte Carlo simulations for inplane and crossplane profiles are <2.6% and 2.5%, respectively.

### 3.B Dynamic Wave Arc (DWA) verification

**Fig. 10:**
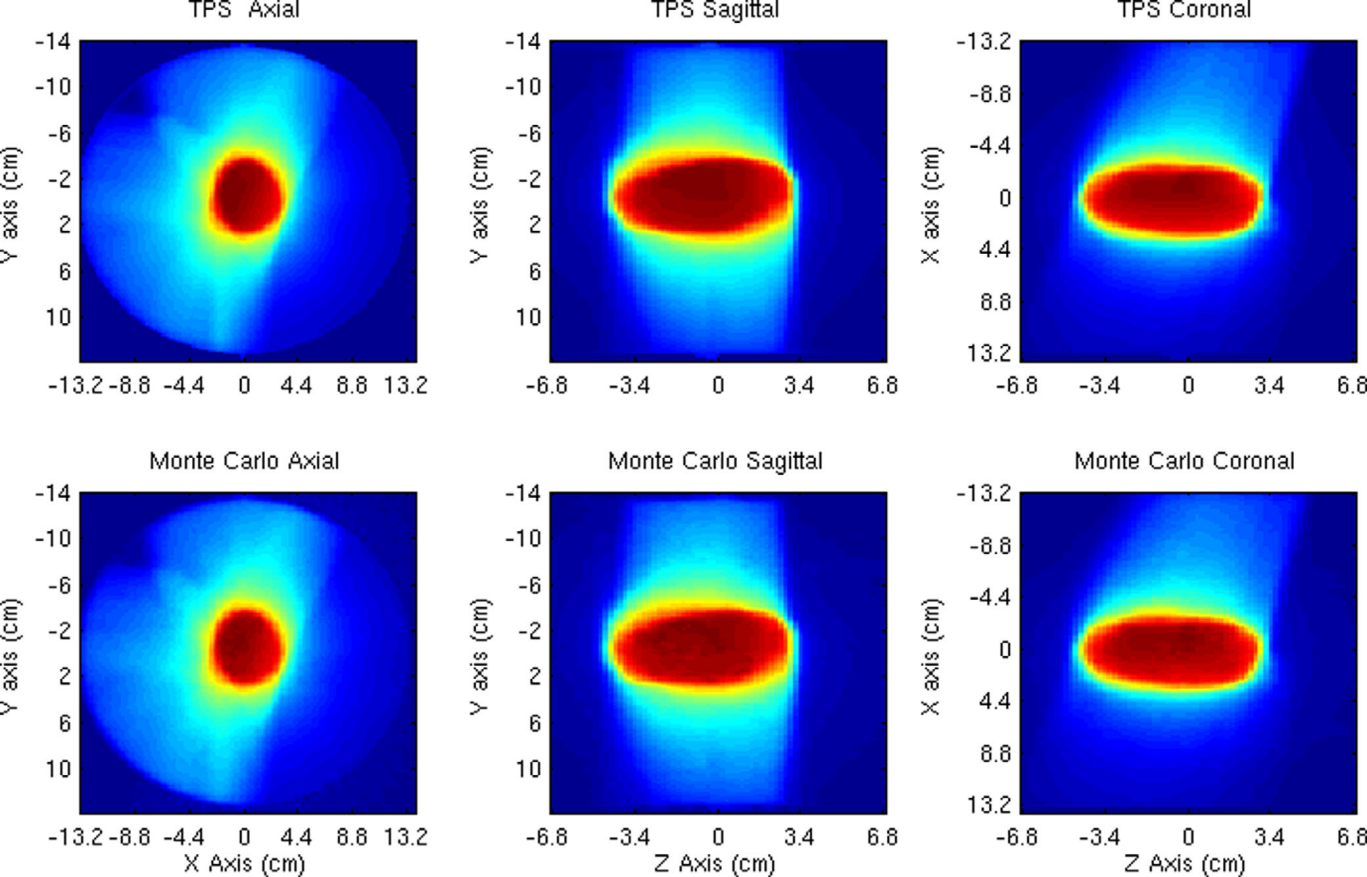
Three‐dimensional dose distributions on a cylinder phantom in three planes shown for RayStation treatment planning system calculations and Monte Carlo simulations.

**Fig. 11:**
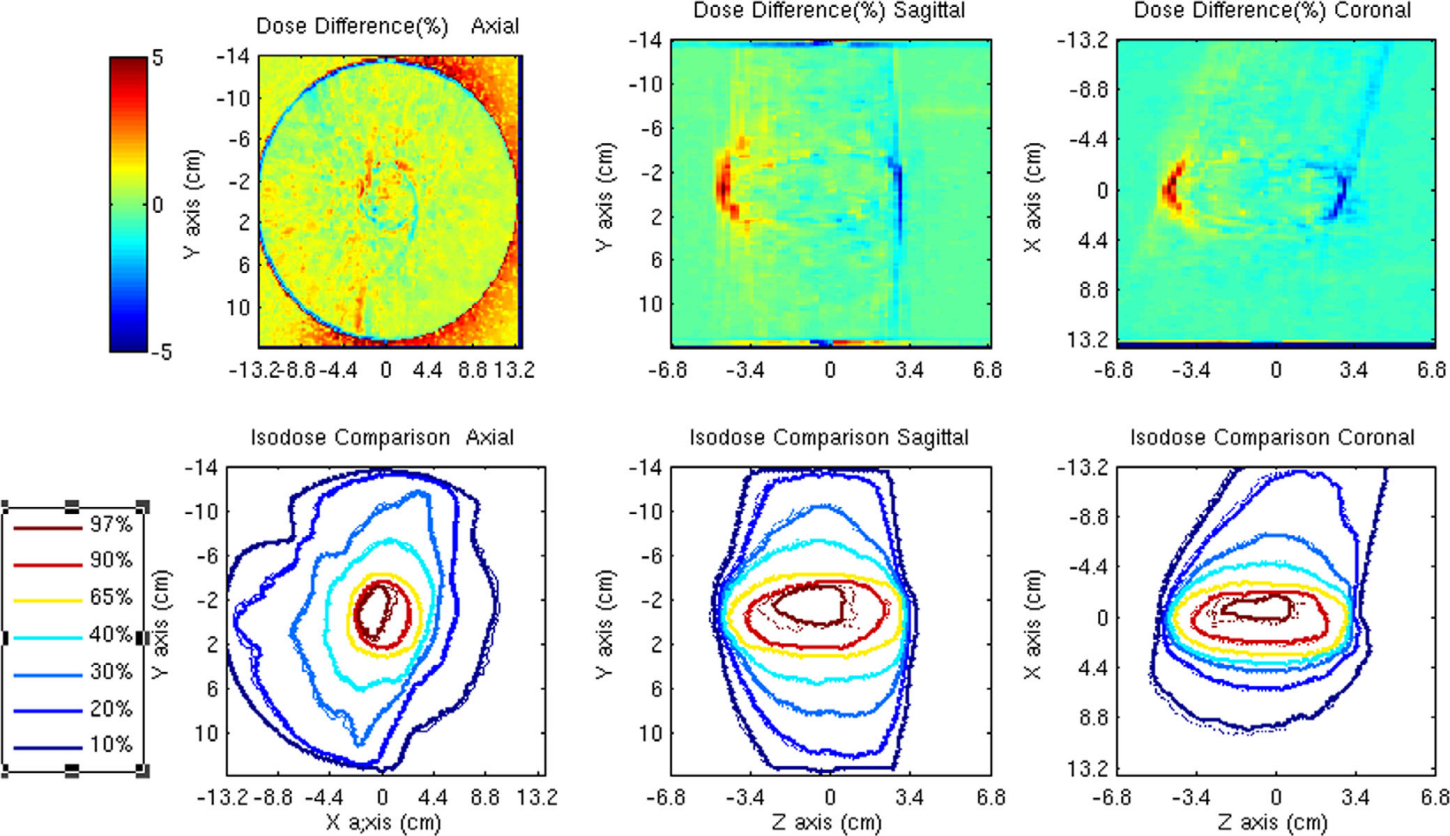
Three‐dimensional dose differences and Isodose overlay RayStation treatment planning system (TPS) calculations and Monte Carlo simulations comparisons on a cylinder phantom in three planes. TPS data (solid line) and Monte Carlo data (dotted line).

The RayStation TPS dose distribution for the DWA Liver SABR plan delivered to the cylindrical uniform QA phantom is compared to Monte Carlo simulations in Fig. 10. The 3D gamma comparison for 3% dose difference relative to max dose ((MC dose − TPS dose)/max dose in TPS) and 3 mm distance to agreement with 30% max dose threshold is 99.3% (Fig. 11). This model has been implemented clinically as a quality assurance tool for the RayStation TPS and has been applied to over 72 patient plan verifications to date. The mean and [min, max] 3D gamma for these patient plans are 98.5% [95.9%, 99.6%]. The 95% confidence interval for the mean value of 3D gamma for those 72 treatment plans is [98.4%, 98.7%].

**Fig. 12:**
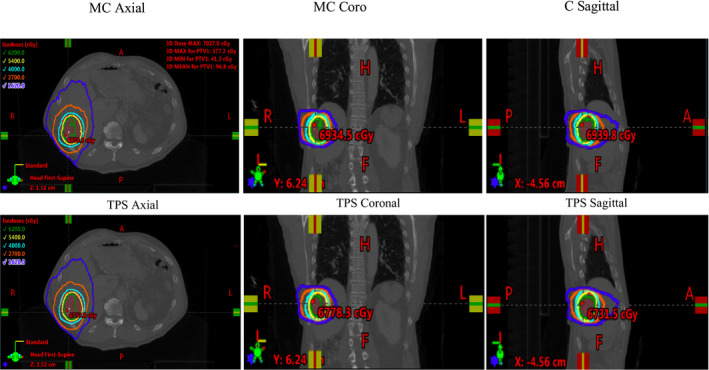
Dose distribution comparison between Monte Carlo and RayStation treatment planning system on a SABR liver patient computed tomography dataset for DWA plan.

**Fig. 13:**
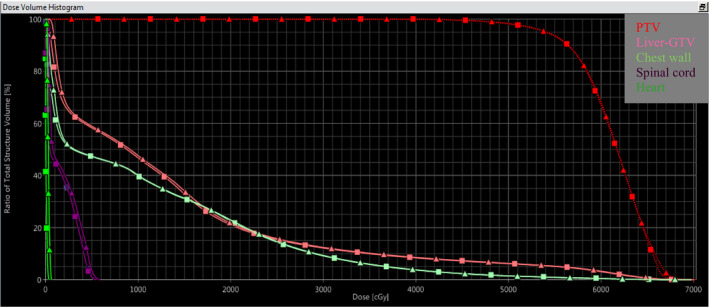
Comparison of planning target volume and organs at risk dose‐volume histograms calculated by treatment planning system (square) and MC (triangle) for DWA plan.

Figure 12 shows the dose distribution calculated by Monte Carlo simulation and RayStation TPS (CCC algorithm) on the patient CT dataset in axial, coronal, and sagittal views. The noncoplanar VMAT trajectory geometry is modeled correctly. PTV and OAR dose–volume histograms are compared in Fig. 13. It should be noted that the RayStation CCC algorithm reports dose as “dose to water,” which is consistent with many commercial treatment planning systems. The Monte Carlo method described here calculates dose to the medium, *D_m_*, directly. This should be considered when making dose comparisons, particularly in bone^31^.

A comparison of dosimetry metrics for OARs and PTV is listed in Table 2. PTV coverage by the 100% prescription isodose (54 Gy) is agreed within 0.1%. OAR maximum (to 0.035 cc volume) and mean doses agreed within 1 Gy.

**Table 2:** Comparison of dosimetry metrics for (a) PTV and (b) OAR calculated by MC and RayStation for a liver SABR treatment plan using dynamic, non‐coplanar trajectories (dynamic wave VMAT arc).

PTV	RS TPS (CCC algorithm)	MC simulation
V_54Gy (100%)_	95.3%	95.2%
mean dose (Gy)	61.1	61.3
D_0.035cc_ (Gy)	67.8	70.3

OARs	RS TPS (CCC algorithm)/Gy	MC simulation/Gy
Liver (minus) GTV/mean dose	13.3	13.7
Chest‐wall/D_0.035cc_	66.9	67.9
Spinal cord/D_0.035cc_	5.5	5.9
Heart/D_0.035cc_	0.4	0.7

## 4 DISCUSSION

The Brainlab Vero4DRT linear accelerator was modeled successfully with Monte Carlo simulation code using EGSnrc (BEAMnrc/DOSXYZnrc) employing source 20 “moving source” and a significantly modified SYNCVMLC dynamic MLC model. Simulated simple radiation benchmark doses in a water phantom were compared to measured commissioning data to allow for fine tuning of the Monte Carlo model. The PDD differences between the MC simulation and the water‐tank measurements were minimized with an incident electron energy of 5.9 MeV in the Monte Carlo simulation, which is closer to nominal energy of 6.0 MeV. The calculated beam profile with an electron beam gaussian width of 1.9 mm demonstrated the closest agreement with measurements as determined by calculating the percentage difference between data values in this region. Ishihara et al.^18^ reported the energy of the incident electron beam and gaussian intensity profile (FWHM) of 6.7 MeV and 1.0 mm, respectively.

The obtained results cannot be directly compared with other types of accelerators by different manufactures because each accelerator has vendor‐specific components such as target, flattening filter, etc.

The maximum difference between measurement and Monte Carlo simulations is <1.64% for the descending part of the 15 × 15 cm^2^ field size PDD with 94.7% of points beyond the buildup demonstrating an agreement of <=1.0%. Ishihara et al. reported that the simulated PDD beyond the buildup region for their model had an agreement of <1.0% for 15 × 15 cm^2^ field size in a water phantom.

The EPID measured and Monte Carlo simulated leakage along the path perpendicular to MLC has been compared. The average EPID measured transmission value is 0.17%. The MC calculated average transmission is 0.18%. Ishihara et al^18^ reported the simulated and the measured leaf leakage. From the MLC simulation result, the interleaf leakage was 0.13% and, in the measurement, the interleaf leakage value was 0.11%. This model can provide MC simulations of clinical, VMAT noncoplanar trajectories (DWA) in addition to more conventional treatment techniques available on the Vero4DRT. Ishihara et al have previously modeled static‐gantry and ring geometries (3DCRT, static‐field IMRT).^18^ Modifications to the Ishihara MC MLC model described in this manuscript now allows for dynamic, simultaneous gantry, ring, and MLC calculations in a single simulation. This will be an invaluable quality assurance tool when combined with a rigorous general machine quality assurance program. This will relieve the need to perform on‐linac patient‐specific phantom measurements for these complex wave arc plans prior to starting treatment. This model can be easily transition to patient‐specific dose reconstructions on a per‐fraction basis using machine delivery log files. Currently, using 256 CPU, a multiarc, noncoplanar simulation on patient geometries takes approximately 23 min.

## 5 CONCLUSIONS

The authors have presented an efficient and accurate Monte Carlo model of the Vero4DRT radiotherapy linac. This model has been implemented clinically as a quality assurance tool for the RayStation TPS and has been applied to over 72 patient plan verifications to date.

## CONFLICT OF INTEREST

The authors have no relevant conflict of interest to disclose.

## AUTHOR CONTRIBUTIONS

All authors reviewed the results and approved the final version of the manuscript. AB, IAP, and MR were involved in study conception and design. MR, AB, IAP, FR, YI, and MN were involved in Monte Carlo data. AB, AM, and EG were involved in treatment planning system data. MR and AB were involved in data analysis and manuscript preparation. All authors were involved in manuscript revision/editing.
